# Assessment of the prognostic factors in patients with pulmonary carcinoid tumor: a population‐based study

**DOI:** 10.1002/cam4.1515

**Published:** 2018-05-07

**Authors:** Yiwei Huang, Xiaodong Yang, Tao Lu, Ming Li, Mengnan Zhao, Xingyu Yang, Ke Ma, Shuai Wang, Cheng Zhan, Yu Liu, Qun Wang

**Affiliations:** ^1^ Department of Thoracic Surgery Zhongshan Hospital Fudan University No. 180, Fenglin Road Shanghai 200032 China; ^2^ Eight‐year Program Clinical Medicine Shanghai Medical College Fudan University Shanghai 200032 China

**Keywords:** Carcinoid tumor, chemotherapy, nomogram, prognostic factors, radiotherapy, surgical treatment

## Abstract

Previous studies have identified potential risk factors for pulmonary carcinoid tumors and evaluated the effect of various treatments; however, the results were not entirely consistent. We conducted a population‐based study to further explore relevant prognostic issues. We extracted cases with pulmonary carcinoid tumors from the Surveillance Epidemiology and End Results database. Cox proportional hazard regression was utilized to identify potential significant risk factors, which helped establish a nomogram for predicting long‐term survival. Survival analysis and a competing risk study were conducted to evaluate the value of different surgical approaches. There were 7057 cases included in the study. Univariate and multivariate analyses showed that age, sex, tumor size, stage, histology, surgical type, chemotherapy, and radiation therapy were all significant prognostic factors. A nomogram with good accuracy for predicting 10‐year survival was formulated. Furthermore, patients who had undergone surgery had a significantly better survival than those who did not undergo surgery. There was no significant prognostic difference between lobectomy and sublobectomy stratified by tumor stage; however, lobectomy was associated with a significantly better survival in atypical tumors, especially those with regional disease. Our research identified possible risk factors in a large cohort and constructed a nomogram to visually predict 10‐year survival of pulmonary carcinoid tumors. We showed that lobectomy and sublobectomy should be considered as the mainstay of treatment, especially lobectomies for atypical tumor.

## Introduction

A pulmonary carcinoid tumor is a neuroendocrine tumor with indolent behavior. Pulmonary carcinoid tumors have a relatively low incidence, ranging from 1.0% to 2.2%, of all lung cancers, but could lead to death in 5–25% of patients with this disease according to previous reports 1, 2. Pulmonary carcinoid tumors are generally classified into two types based on histological features as follows: typical (TC) and atypical (AC) 3. Compared with TCs, patients with ACs were more likely to have lymph node metastases and a worse prognosis 4, 5, 6. It is widely accepted that surgical resection is the mainstay of treatment for pulmonary carcinoid tumors 7, but controversy still exists with respect to the optimal extent of surgery. Limited evidence involving small cohorts has reported the prognostic values of systemic chemotherapy or radiation therapy for pulmonary carcinoid tumors, but the results were not consistent 8, 9. More detailed subgroup analyses associated with tumor stage or histology are also limited.

In this study, we extracted data from the Surveillance Epidemiology and End Results (SEER) database and identified potential prognostic factors for pulmonary carcinoid tumors in a very large population‐based cohort. A nomogram was constructed to visually predict the long‐term cancer‐specific survival based on the multivariate prognostic model of this cohort. Assessment of the efficacy of different surgical approaches was performed. We also performed subgroup analyses stratified by tumor stage and histological type to further explore the prognostic significance. Competing risk analysis was utilized as an important supplement to the analysis.

## Materials and Methods

**Figure 1:**
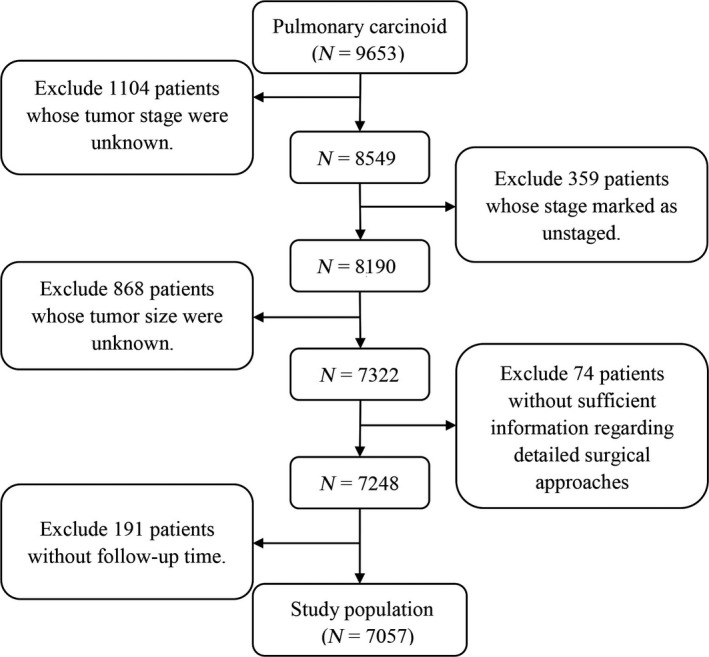
The flow diagram of the selection process for the study cohort.

All patient data were extracted from the SEER database using the SEER*stat software (version 8.3.4; http://seer.cancer.gov/seerstat/). The SEER database updates annually, including information of survival and follow‐up. In this study, the latest patient information was updated in November 2016, and the whole database released in April 2017. The median follow‐up time was 75 months (25%, 75% percentile: 32 to 133 months). And the average follow‐up time was 90.5 months. The selection process was as follows (Fig. 1). Patients who had pulmonary carcinoid tumors (ICD‐O‐3: 8240 and 8249) as their first primary tumor between 1973 and 2014 were identified. Patients diagnosed before 1988 were excluded because their records did not contain sufficient information for staging in the SEER database. Then, patients with unknown surgical approach, tumor size, or follow‐up time were also excluded. Patients who did not undergo surgery or underwent surgical resection (lobectomy, sublobectomy, or pneumonectomy) were included in the analysis. The baseline characteristics of all patients were collected, including race, histological type, sex, age at diagnosis, tumor stage (SEER historical stage A), tumor size, and subsequent treatment. Due to the incompleteness of tumor stage for this disease in the database, the variable “SEER historical stage A” was adopted as in similar previous studies. The tumors were mainly classified into the following three types: localized (confined to the origin), regional (beyond the surroundings and/or regional lymph node metastases), and distant (spread into distant organs, tissues, or distant lymph nodes). In this study, we adopted cancer‐specific survival (CSS) and overall survival (OS) from the database as the survival outcomes. Deaths that were attributed to pulmonary carcinoid tumors were regarded as cancer‐specific deaths, and other causes of deaths were considered censoring events 10.

All statistical analyses were performed using SPSS 24.0 (IBM, Inc., Armonk, NY), R software (version 3.3.3; R Foundation for Statistical Computing, Vienna, Austria), and Prism 6 software (Version 6.01; GraphPad Software Inc., CA). Univariate and multivariate Cox proportional hazard models were utilized to identify potential significant prognostic factors for the entire cohort. The final model was backward stepdown selected with the Akaike information criterion. A nomogram was formulated based on the final model and was evaluated by the concordance index (C‐index) and the calibration curve. The clinicopathological variables between groups were analyzed using the Pearson chi‐square test or the Student *t‐*test. Survival curves were performed using the Kaplan–Meier method, and a log‐rank test was adopted to compare the curves. Competing risk analysis was also performed, as in previous studies 11, 12, as other causes of death were competing outcomes for cancer‐specific deaths. Cumulative incidence functions were calculated, and Gray's test was used to compare the estimated cumulative incidence curves. A two‐tailed *P*‐value <0.05 was considered statistically significant. The Institutional Review Committee of Zhongshan Hospital (Fudan University, Shanghai, China) approved this study to be exempted research.

## Results

We have identified 12,607 cases with pulmonary carcinoid tumors, accounting for approximately 1.09% of 1,152,689 cases of lung cancer from the SEER database between 1973 and 2014. Following the inclusion criteria described above, a total of 7057 patients were finally extracted for this study. The baseline characteristics of all patients are shown in Table 1. The 10‐year cancer‐specific survival rate was 88.1%. There were 6554 and 503 patients with TC and AC tumors, respectively. The corresponding 10‐year cancer‐specific survival rate was 89.8% and 59.9%, respectively. Patients who underwent lobectomies accounted for 61.6% of the entire cohort. Chemotherapy and radiation therapy were adopted in 4.5% and 4.3% of the study population during the entire treatment course, respectively.

**Table 1:** Patient characteristics and results of univariate and multivariate analyses of cancer‐specific survival in the cohort

		Univariate			Multivariate		
Characteristics	*N*	HR	95% CI	*P*‐value	HR	95% CI	*P*‐value
Total	7057						
Age at diagnosis	57.9 ± 15.3	1.05	1.04–1.05	<0.001	1.04	1.04–1.05	<0.001
Gender							
Male	2333			<0.001			<0.001
Female	4724	1.52	1.29–1.80		1.36	1.14–1.61	
Race							
White	6354			0.270			
Black	474						
Other	229						
Histological type							
Typical carcinoid	6554			<0.001			<0.001
Atypical carcinoid	503	4.89	4.02–5.96		2.45	1.97–3.04	
Stage							
Localized	5244			<0.001			<0.001
Regional	1308	3.43	2.86–4.11	<0.001	2.52	2.08–3.06	<0.001
Distant	505	14.42	11.95–17.40	<0.001	4.57	3.63–5.75	<0.001
Radiotherapy							
No	6741			<0.001			<0.001
Yes	316	8.97	7.42–10.84		1.91	1.53–2.39	
Chemotherapy							
No	6752			<0.001			<0.001
Yes	305	8.71	7.19–10.56		1.61	1.26–2.06	
Surgery							
No	828			<0.001			<0.001
Lobectomy	4347	0.11	0.09–0.13	<0.001	0.40	0.32–0.50	<0.001
Sublobar resection	1537	0.10	0.08–0.13	<0.001	0.40	0.30–0.53	<0.001
Pneumonectomy	345	0.20	0.15–0.28	<0.001	0.54	0.38–0.76	<0.001
Tumor size (cm)	2.4	1.03	1.03–1.04	<0.001	1.02	1.01–1.02	<0.001

HR, hazard ratio; CI, confidence interval.

Univariate analyses of CSS showed that the patient's age (*P *< 0.001), sex (*P* < 0.001), tumor size (*P* < 0.001), tumor stage (*P* < 0.001), histology type (*P* < 0.001), surgical type (*P* < 0.001), chemotherapy (*P* < 0.001), and radiation therapy (*P* < 0.001) were all significant predictors of the outcome, which were similar to the results of univariate analyses for OS. Furthermore, all of the aforementioned variables remained as independent prognostic factors in the multivariate analyses for CSS and OS (Tables 1 and S1).

**Figure 2:**
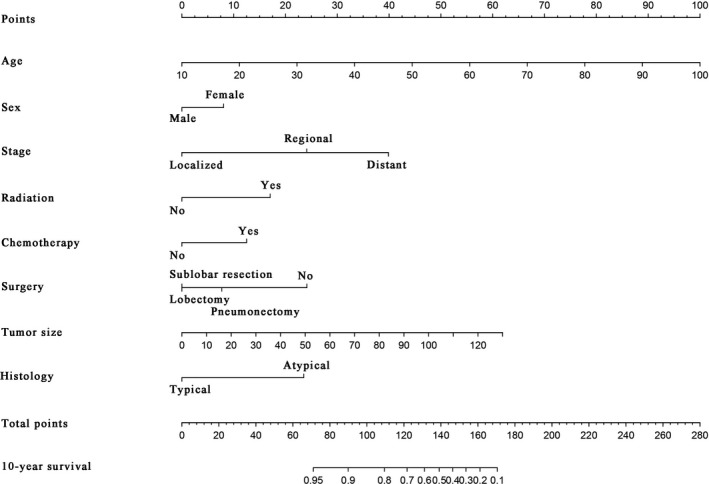
A nomogram for prediction of 10‐year cancer‐specific survival of patient with pulmonary carcinoid tumor.

A nomogram was then established based on all significant risk factors identified by the multivariate analyses for predicting 10‐year CSS (Fig. 2). We obtained the points for each risk factor listed in the nomogram and then summed the points. The 10‐year CSS rate for pulmonary carcinoid tumors was estimated using the bottom point scale in the nomogram. The C‐index for prediction was 0.863, and thus, the prediction accuracy of this nomogram was quite good. The calibration plot of this nomogram based on 1000 times bootstrap resampling is also provided in Figure S1.

Table S2 shows the distribution of variables across the TC and AC groups. Patients with ACs tended to be older (*P* < 0.001) and with more advanced diseases (*P* < 0.001), as well as had a trend toward a larger tumor (*P* < 0.001) and a larger proportion of patients who received radiotherapy or chemotherapy (*P* < 0.001). The 5‐year CSS and 10‐year CSS were significantly lower than patients with TCs.

**Figure 3:**
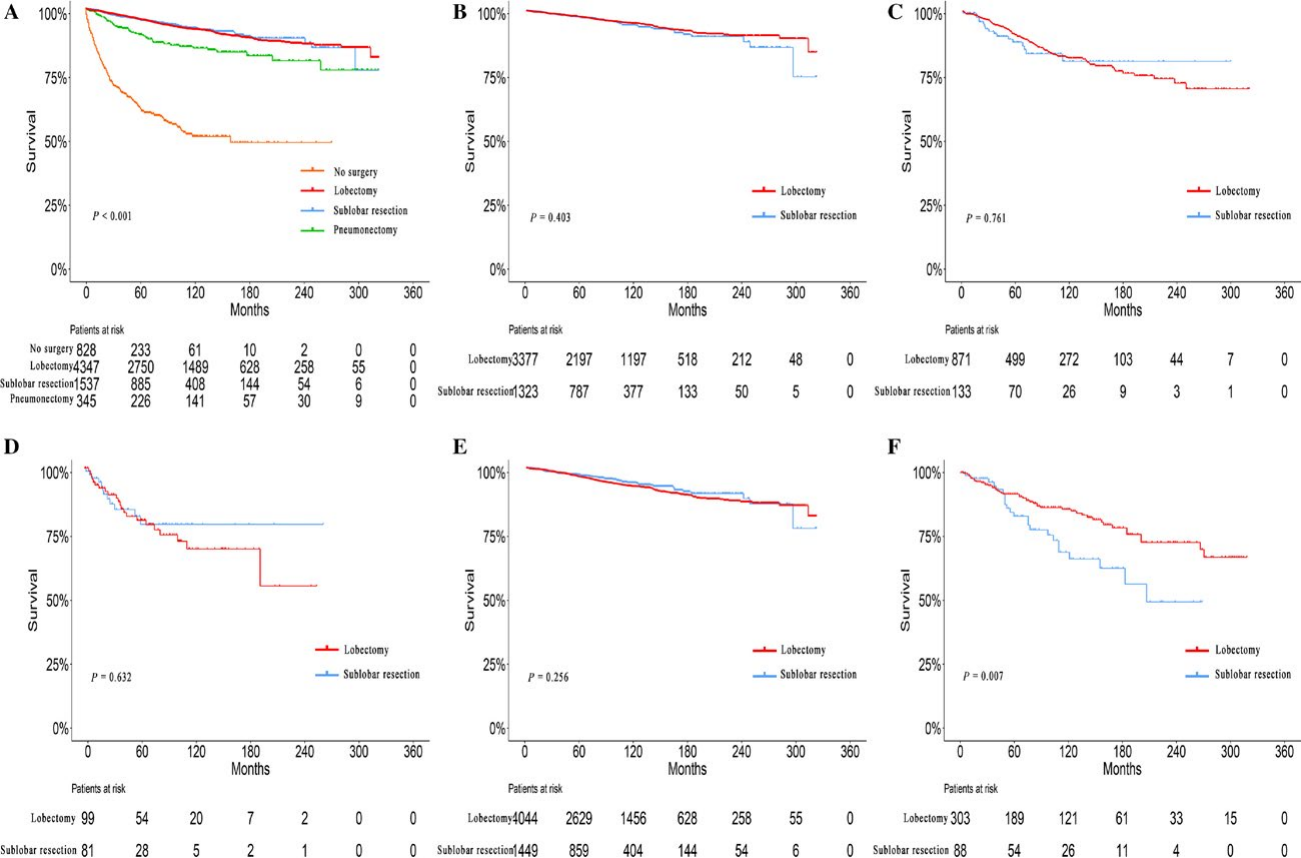
(A) Survival analyses for patients with various surgery and without surgery. Survival analyses for patients with lobectomy and sublobar resection stratified by tumor stage and by tumor histology. (B) Localized (10‐year survival rate: 95.3% vs. 94.7%). (C) Regional (10‐year survival rate: 82.4% vs. 81.2%). (D) Distant (10‐year survival rate: 69.6% vs. 78.8%). (E) Typical carcinoid (10‐year survival rate: 99.6% vs. 94.7%). (F) Atypical carcinoid (10‐year survival rate: 72.5% vs. 49.1%).

Survival curves were utilized to compare the efficacy of surgical resections of different extents (Fig. 3A). Patients who underwent surgeries had a significantly better CSS than patients who did not undergo surgery (*P* < 0.001). Pneumonectomy conferred a significantly worse prognosis than lobectomy and sublobectomy (*P* < 0.001).

**Figure 4:**
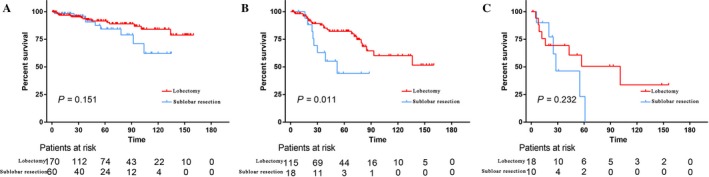
Survival analyses for patients with atypical carcinoids received lobectomy and sublobar resection stratified by tumor stage: (A) Localized (10‐year survival rate: 84.1% vs. 62.2%). (B) Regional (10‐year survival rate: 60.2% vs. 44.1%). (C) Distant (10‐year survival rate: 33.7% vs. 0.0%).

The baseline characteristics of the patients who underwent lobectomy and sublobar resection are listed in Table S3. Patients who received sublobar resection were more likely to be older (*P* < 0.001), had a smaller tumor (*P* < 0.001), and were in more advanced stages (*P* < 0.001). Moreover, no significant prognostic difference existed between lobectomy and sublobar resection (*P* = 0.790). There was no significant difference in CSS between lobectomy and sublobectomy when stratified by tumor stage (*P* = 0.403, 0.761, and 0.632, respectively; Fig. 3B–D). However, lobectomy was associated with significantly better survival than sublobar resection in AC tumors (*P* = 0.256), but not in TC tumors (*P* = 0.007; Fig. 3E–F). Competing risk analyses also revealed similar results (Fig. S2). As for patients with AC tumors, further analysis showed that lobectomy conferred a significantly better prognosis than sublobar resection in patients with regional disease (*P* = 0.011; Fig. 4). In addition, lobectomy also showed advantages in prognosis for localized disease, although without statistical significance (Fig. 4).

## Discussion

A pulmonary carcinoid tumor is a neuroendocrine tumor, which has a relatively indolent behavior compared with lung adenocarcinomas or squamous cell carcinoma; however, the optimal treatment for pulmonary carcinoid tumors, including surgical approaches, is controversial despite based on previous studies. In the current study, we extracted a large number of cases from a population‐based database. We identified possible independent prognostic factors and established a nomogram to accurately predict long‐term survival in patients with pulmonary carcinoid tumors. Survival analyses were performed to evaluate the potential prognostic significance of different surgical procedures. More detailed assessments were conducted in subgroups stratified by tumor stage using competing risk analyses.

For carcinoid tumor, one of the most important prognostic factors is a histological subtype. As TC and AC have very different clinical features, histology classification is very essential 13. Our results showed that patients with AC were in more advanced stages, which correlated with previous findings. For most TC tumors, surgical resection is the optimal treatment 14, 15. Previous studies have indicated that surgically resected TCs were associated with a significantly better prognosis than ACs, which was consistent with our findings in this study 16, 17.

Surgical resection of carcinoid tumors is recommended by most surgeons 14, 18; however, Raz et al. 19 observed that the 5‐year survival rate was also quite high in patients managed nonsurgically. Our research revealed that patients who underwent surgery had a significantly better CSS than patients who did not undergo surgery. Lobectomy is still the mainstay surgical approach for nonsmall cell lung cancer, and sublobar resection is considered and performed in select patients with more and more supporting evidence. In the current study, pneumonectomy was shown to have a significantly worse survival than lobectomy and sublobectomy, which may be due to the massive loss of pulmonary function. Yendamuri et al. 20 reported that lobectomy is not a negative prognostic factor for patients with TC tumors and a practicable alternative to more extensive resection. Furthermore, it was also reported that the OS of sublobar resection was comparable to lobectomy in patients with cT1aN0M0 TCs based on the National Cancer Data Base 21. Similarly, it was deemed to be advisable to implement anatomical sublobar resection for patients with small peripheral TCs 16; however, some surgeons believe that even TC tumors have the potential for distant metastasis, and more aggressive resection should be planned due to the unpredictable nature of the disease. For AC tumors, lobectomy with mediastinal lymph node resections is advocated 22, 23. In the current study, we showed that there was no significant prognostic difference between lobectomy and sublobar resection stratified by tumor stage; however, lobectomy may confer a significantly better survival in patients with AC tumors, but not TC tumors. More detailed studies regarding recurrence outcomes are also needed to explore the potential difference between lobectomy and sublobar resection, especially when stratified by tumor histology.

Currently, the efficacy of chemotherapy for lung carcinoid tumors remains controversial 24. Daniel et al. 25 considered that adjuvant chemotherapy did not improve the survival of patients who underwent lobectomy for TCs with lymph node metastases. In contrast, Caplin et al. 26 reported that some chemotherapeutics are effective for unresectable tumors, especially low‐grade TCs and ACs. The effect of radiotherapy on pulmonary carcinoid tumors remains to be studied. Colaco et al. 8 argued that stereotactic body radiotherapy is beneficial for inoperable patients, which could control disease progression and reduce toxicity. In this study, multivariate analyses showed that radiotherapy and chemotherapy were negative prognostic factors.

However, further studies with larger cohort were needed.

The data of additional treatments such as the utilization of somatostatin analogues (SSA) and peptide receptor radionuclide therapy (PRRT) were not provided by the SEER. Previous study showed that interferon and octreotide may provide effective symptomatic relief, but only in 15% of cases 27. The role of systemic treatment of metastatic disease can be divided into four categories: PRRT, targeted therapy, chemotherapy, or SSA therapy 28. However, the specific impact of these treatments on prognosis remains to be further explored.

This retrospective analysis included a large study cohort from the SEER database. We analyzed the prognostic value of different surgical approaches for lung carcinoid tumor. In addition, a nomogram for predicting long‐term CSS was established. There are some limitations that should be mentioned. Owing to the limitations of the SEER database for lung carcinoid tumors, the TNM stage records were incomplete. Detailed information regarding chemotherapy and radiotherapy could not be provided. Further randomized controlled trials should be conducted with more clinicopathological characteristics, which may validate and optimize our nomogram.

## Conclusions

Our study showed the potential risk factors for the prognosis of lung carcinoid tumors, and a nomogram predicting long‐term survival was established. We found that lobectomies and sublobar resections should be considered as the mainstay of treatment for carcinoid tumors. With respect to AC tumors, lobectomy could lead to a significantly better prognosis.

## Conflict of Interests

The authors have no conflict of interests to declare.

## Supporting information


**Figure S1.** Calibration curve of the nomogram predicting 10‐year cancer‐specific survival of patients with pulmonary carcinoid tumor.

**Figure S2.** Competing risk analyses for patients with lobectomy and sublobar resection stratified by tumor stage and by tumor histology (A) Localized. (B) Regional. (C) Distant. (D) Typical carcinoid. (E) Atypical carcinoid. 1: cancer‐specific death; 2: other death.

**Table S1.** Patient characteristics and analyses based on overall survival status.
**Table S2.** Baseline characteristics of patients with typical and atypical carcinoids.
**Table S3.** Baseline characteristics of patients with lobectomy and sub‐lobar resection.
