# Supplementary figures and images for: Assessment of the prognostic factors in patients with pulmonary carcinoid tumor: a population‐based study
Source: Cancer Med. 2018 May 7;7(6):2434–41. doi: 10.1002/cam4.1515 (PMC6010747; doi:10.1002/cam4.1515)

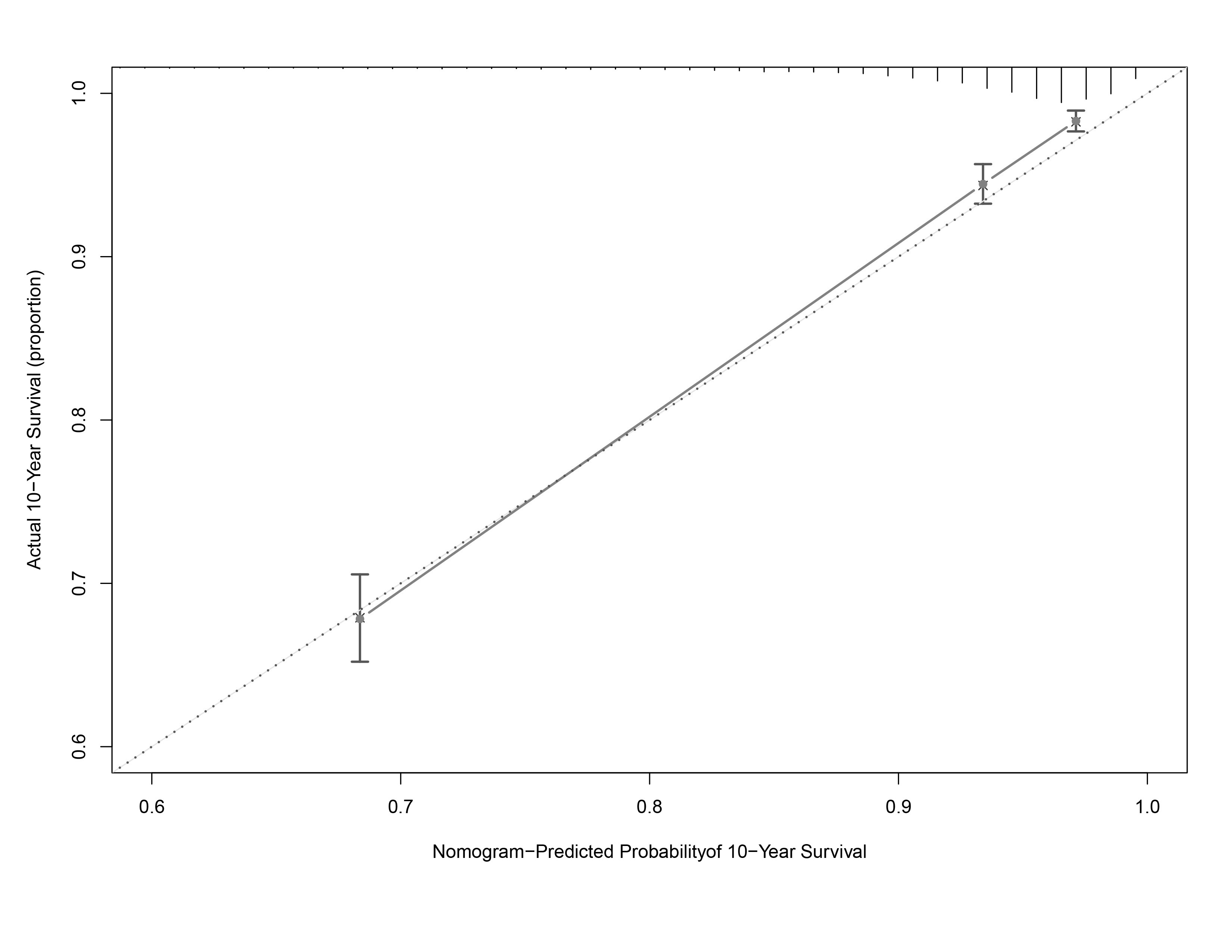

Supplement: Supplementary file 1 — Figure S1. Calibration curve of the nomogram predicting 10‐year cancer‐specific survival of patients with pulmonary carcinoid tumor. [file CAM4-7-2434-s001.tif]

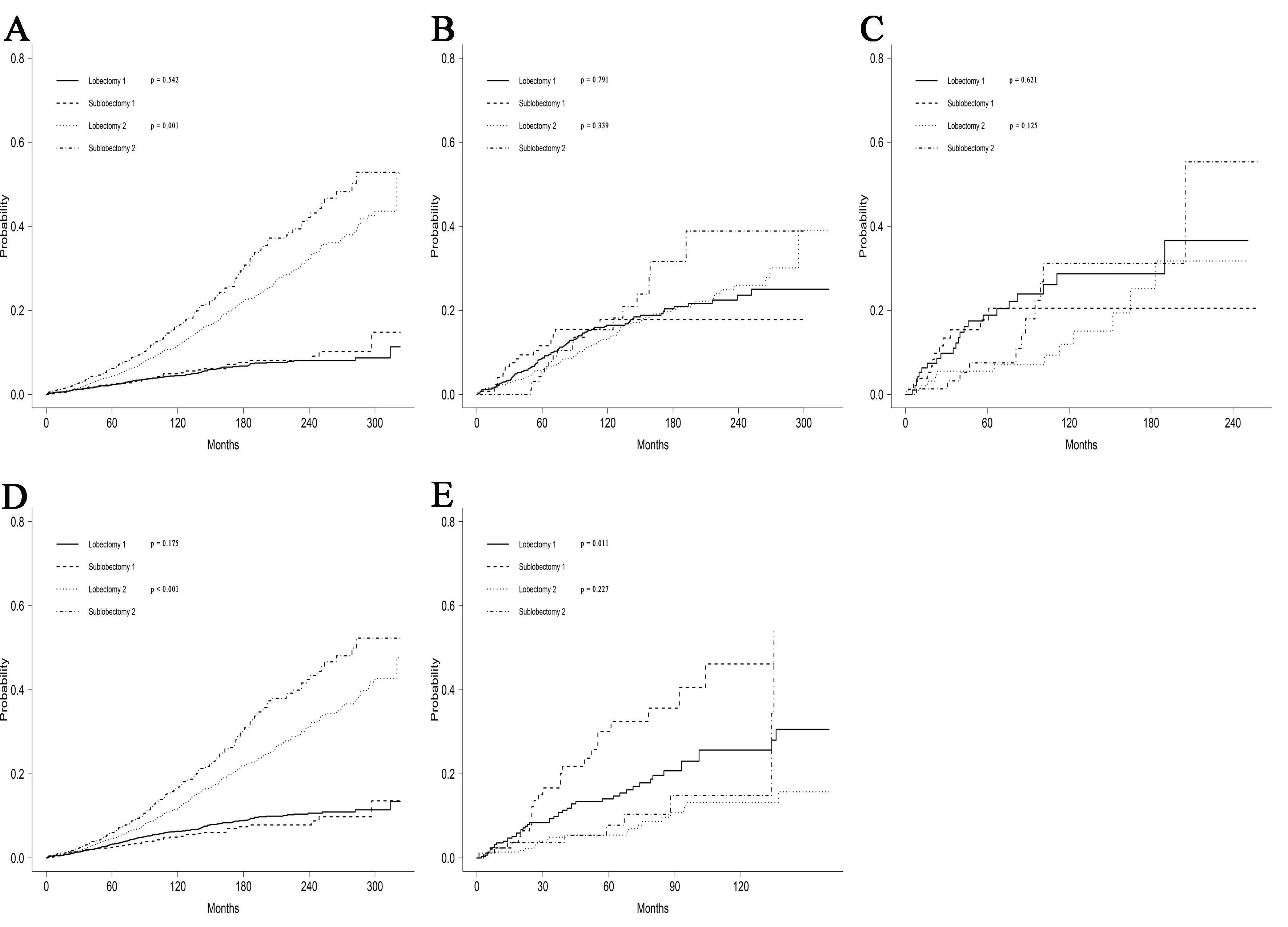

Supplement: Supplementary file 2 — Figure S2. Competing risk analyses for patients with lobectomy and sublobar resection stratified by tumor stage and by tumor histology (A) Localized. (B) Regional. (C) Distant. (D) Typical carcinoid. (E) Atypical carcinoid. 1: cancer‐specific death; 2: other death. [file CAM4-7-2434-s002.tif]
