# Supplementary material for: Assessment of the prognostic factors in patients with pulmonary carcinoid tumor: a population‐based study
Source: Cancer Med. 2018 May 7;7(6):2434–41. doi: 10.1002/cam4.1515 (PMC6010747; doi:10.1002/cam4.1515)
Supplement: Supplementary file 3 — Table S1. Patient characteristics and analyses based on overall survival status. Table S2. Baseline characteristics of patients with typical and atypical carcinoids. Table S3. Baseline characteristics of patients with lobectomy and sub‐lobar resection. [file CAM4-7-2434-s003.docx]

**Supplementary table 1**

Patient characteristics and analyses based on overall survival status.

| Characteristics | N | univariate | | | multivariate | | |
| --- | --- | --- | --- | --- | --- | --- | --- |
|  |  | HR | 95%CI | p-value | HR | 95%CI | p-value |
| Total | 7057 |  |  |  |  |  |  |
| Age at diagnosis | 57.9 | 1.07 | 1.06-1.07 | <0.001 | 1.06 | 1.06-1.07 | <0.001 |
| Gender |  |  |  | 0.009 |  |  | 0.038 |
| Male | 2333 |  |  |  |  |  |  |
| Female | 4724 | 1.15 | 1.03-1.27 |  | 1.12 | 1.01-1.24 |  |
| Race |  |  |  | 0.030 |  |  |  |
| White | 6354 |  |  |  |  |  |  |
| Black | 474 | 1.20 | 1.01-1.44 | 0.042 |  |  |  |
| Other | 229 | 1.28 | 0.98-1.70 | 0.072 |  |  |  |
| Histologic type |  |  |  | <0.001 |  |  | <0.001 |
| Typical carcinoid | 6554 |  |  |  |  |  |  |
| Atypical carcinoid | 503 | 3.00 | 2.55-3.53 |  | 1.93 | 1.63-2.30 |  |
| Stage |  |  |  | <0.001 |  |  | <0.001 |
| Localized | 5244 |  |  |  |  |  |  |
| Regional | 1308 | 1.67 | 1.49-1.88 | <0.001 | 1.58 | 1.40-1.79 | <0.001 |
| Distant | 505 | 5.97 | 5.20-6.86 | <0.001 | 2.38 | 2.02-2.81 | <0.001 |
| Radiotherapy |  |  |  | <0.001 |  |  | <0.001 |
| No | 6741 |  |  |  |  |  |  |
| Yes | 316 | 4.98 | 4.27-5.80 |  | 1.84 | 1.54-2.20 |  |
| Chemotherapy |  |  |  | <0.001 |  |  | <0.001 |
| No | 6752 |  |  |  |  |  |  |
| Yes | 305 | 4.51 | 3.83-5.30 |  | 1.74 | 1.43-2.10 |  |
| Surgery |  |  |  | <0.001 |  |  | <0.001 |
| No | 828 |  |  |  |  |  |  |
| Lobectomy | 4347 | 0.15 | 0.13-0.17 | <0.001 | 0.46 | 0.40-0.54 | <0.001 |
| Sub-lobar resection | 1537 | 0.19 | 0.16-0.22 | <0.001 | 0.51 | 0.43-0.60 | <0.001 |
| Pneumonectomy | 345 | 0.17 | 0.13-0.21 | <0.001 | 0.57 | 0.44-0.73 | <0.001 |
| Tumor size (cm) | 2.4 | 1.02 | 1.01-1.02 | <0.001 | 1.01 | 1.01-1.02 | <0.001 |

HR: hazard ratio; CI: confidence interval.

**Supplementary table 2**

Baseline characteristics of patients with typical and atypical carcinoids.

| Factors | N | Typical | Atypical | p-value |
| --- | --- | --- | --- | --- |
| Total | 7057 | 6554 | 503 |  |
| Age at diagnosis | 57.9±15.3 | 57.6±15.4 | 60.7±13.4 | <0.001 |
| Gender |  |  |  | 0.089 |
| Male | 2333 | 2184(33.3%) | 149(29.6%) |  |
| Female | 4724 | 4370(66.7%) | 354(70.4%) |  |
| Race |  |  |  | 0.627 |
| White | 6354 | 5895(89.9%) | 459(91.3%) |  |
| Black | 474 | 445(6.8%) | 29(5.8%) |  |
| Other | 229 | 214(3.3%) | 15(3.0%) |  |
| Stage |  |  |  | <0.001 |
| Localized | 5244 | 4998(76.3%) | 246(48.9%) |  |
| Regional | 1308 | 1138(17.4%) | 170(33.8%) |  |
| Distant | 505 | 418(6.4%) | 87(17.3%) |  |
| Radiotherapy |  |  |  | <0.001 |
| No | 6741 | 6316(96.4%) | 425(84.5%) |  |
| Yes | 316 | 238(3.6%) | 78(15.5%) |  |
| Chemotherapy |  |  |  | <0.001 |
| No | 6752 | 6362(97.1%) | 390(77.5%) |  |
| Yes | 305 | 192(2.9%) | 113(22.5%) |  |
| Surgery |  |  |  | <0.001 |
| No | 828 | 742(11.3%) | 86(17.1%) |  |
| Lobectomy | 4347 | 4044(61.7%) | 303(60.2%) |  |
| Sub-lobar resection | 1537 | 1449(22.1%) | 88(17.5%) |  |
| Pneumonectomy | 345 | 319(4.9%) | 26(5.2%) |  |
| Tumor size (cm) | 2.4 | 2.3±1.4 | 3.0±1.9 | <0.001 |
| 5-years CSS |  | 93.6±0.3% | 72.3±2.4% |  |
| 10-years CSS |  | 89.8±0.5% | 59.9±3.5% |  |

CSS: cancer-specific survival.

**Supplementary table 3**

Baseline characteristics of patients with lobectomy and sub-lobar resection.

| Factors | N | Lobectomy | Sub-lobar | p-value |
| --- | --- | --- | --- | --- |
| Total | 5884 | 4347 | 1537 |  |
| Age at diagnosis | 56.9±14.8 | 55.6±14.9 | 60.7±13.8 | <0.001 |
| Gender |  |  |  | 0.946 |
| Male | 2021 | 1492(34.3%) | 529(34.4%) |  |
| Female | 3863 | 2855(65.7%) | 1008(65.6%) |  |
| Race |  |  |  | 0.096 |
| White | 5318 | 3933(90.5%) | 1385(90.1%) |  |
| Black | 386 | 272(6.3%) | 114(7.4%) |  |
| Other | 180 | 142(3.3%) | 38(2.5%) |  |
| Stage |  |  |  | <0.001 |
| Localized | 4700 | 3377(77.7%) | 1323(86.1%) |  |
| Regional | 1004 | 871(20.0%) | 133(8.7%) |  |
| Distant | 180 | 99(2.3%) | 81(5.3%) |  |
| Radiotherapy |  |  |  | 0.055 |
| No | 5765 | 4250(97.8%) | 1515(98.6%) |  |
| Yes | 119 | 97(2.2%) | 22(1.4%) |  |
| Chemotherapy |  |  |  | 0.236 |
| No | 5746 | 4239(97.5%) | 1507(98.0%) |  |
| Yes | 138 | 108(2.5%) | 30(2.0%) |  |
| Histologic type |  |  |  | 0.092 |
| Typical carcinoid | 5493 | 4044(93.0%) | 1449(94.3%) |  |
| Atypical carcinoid | 391 | 303(7.0%) | 88(5.7%) |  |
| Tumor size (cm) | 22.2±12.9 | 24.3±13.2 | 16.1±9.7 | <0.001 |
| 5-years CSS |  | 95.9±0.3% | 96.0±0.6% |  |
| 10-years CSS |  | 92.2±0.5% | 92.8±0.9% |  |

CSS: cancer-specific survival.
